# Prehypertension and its associated factors among government employees in Tilottama Municipality of Rupandehi District, Nepal

**DOI:** 10.1371/journal.pone.0338625

**Published:** 2025-12-11

**Authors:** Sheetal Bhandari, Paras Kumar Pokharel, Ashmita Maharjan, Manish Rajbanshi, Kshitij Kunwar, Jwala Subedi, Anup Poudel, Suman Bahadur Singh, Vijay Kumar Khanal, Gyanu Nepal Gurung

**Affiliations:** 1 School of Public Health and Community Medicine, B.P. Koirala Institute of Health Sciences, Dharan, Nepal; 2 Central Department of Public Health, Institute of Medicine, Tribhuvan University, Kathmandu, Nepal; Universiti Putra Malaysia, MALAYSIA

## Abstract

Government employees are vital workforce of the nation comprising individuals in the working age group. These employees have to face an increased workload, limited decision-making scope, lack of support from fellow workers, regular night shifts, limited physical mobility which puts them at increased risk for hypertension. Detecting prehypertension is essential to prevent related complications and reduce risks of future health issues such as an increased risk of developing high blood pressure (hypertension), heart disease, stroke, kidney damage, and other cardiovascular problems. This study aimed to assess the prevalence of prehypertension and its associated factors among the government employees of Tilottama Municipality, Nepal. A cross-sectional study was conducted among 333 government employees from 15 June 2023 to 10 November 2023. A systematic random sampling technique was used to select participants. A structured questionnaire was adapted from the WHO STEPs Survey. Data were cleaned and then exported to IBM SPSS Statistics 20.0 for analysis. Demographic characteristics of respondents were described using descriptive statistics. Multivariate logistic regression was conducted to determine the association between individual characteristics and prehypertension. Statistical significance was set at p-value < 0.05. The mean (±SD) age of the 333 participants was 36.9(±8.89) years. Most of them were females (54.1%). This study found that 29.4% of participants were prehypertensive, and 14.1% were hypertensive. Participant’s age, sex, working department, family history of hypertension, presence of diabetes, BMI, waist-hip ratio, ever-alcohol consumption and duration of service were found to be significantly associated with prehypertension in bivariate analysis. Meanwhile, working department (Adjusted Odds Ratio AOR: 3.2, CI: 1.4–7.0), family history of hypertension (AOR: 1.9, CI: 1.1–3.4), and BMI (AOR: 1.7, CI: 1.0–3.1) were found to be significantly associated with prehypertension. This study concluded that a significant proportion of government employees had prehypertension and hypertension. Targeted health promotion programs should be implemented in non-health departments. Regular hypertension screening program and healthy workplace settings such as healthier food choices, physical activity, stress management techniques, and smoke-free zones should be promoted in each institution, with a focus on hypertension prevention and control for employees.

## Introduction

Hypertension is also known as a silent killer disease affecting mainly the working age group living in developing countries through its devastating burden [1,2]. Prehypertension is defined as a systolic blood pressure of 120–139 mmHg and/or a diastolic blood pressure of 80–89 mmHg [3]. Prehypertension is a preliminary stage of hypertension where an individual can become hypertensive as well as suffer from other CVDs (Cardiovascular Diseases) complications if no preventive measures are taken [4]. Raised blood pressure mostly remains asymptomatic while increasing the risk of heart disease, stroke, and renal failure [5].

It is estimated that 18% of adults in low and middle-income countries (LMICs) are hypertensive, of which two–thirds remain untreated [6]. World Health Organization estimated that two-thirds of hypertensive people live in developing countries including Nepal and India [7]. Africa has the highest prevalence of hypertension (29.6%) followed by the Eastern Mediterranean (26.9%), South East Asia (24.7%), Europe (23.3%), the Western Pacific (18.7%), and America (18.2%) [8]. A survey conducted in the US indicated that 47.4% and 18.4% of female long-term-care nursing assistants had prehypertension and hypertension, respectively [9]. In response WHO adopted the Global NCD (Non-Communicable Disease) Compact 2020–2030 aims to accelerate progress on the prevention and control of NCDs. It seeks to ensure Member States adopt policies and programs that improve NCD outcomes and save the lives of people living with NCDs [10].

In South Asia, Nepal was ranked second in terms of having the highest proportion of hypertensive people (27.3%) after Afghanistan (29%) [8]. Several studies reported the burden of NCDs including hypertension has been increasing in Nepal. The premature mortality due to NCDs has risen from 51% to 71% in the past 10 years [12–14]. It is reported that CVDs are responsible for 30% of all deaths in Nepal [15]. A nationally based report in 2024 revealed that one in every four adults had increased blood pressure (24.5%) and body mass index (24.3%). A study in Bangladesh reported a high burden of hypertension among government employees. Employees with average or low job satisfaction were more likely to have hypertension compared to those fully satisfied with their jobs. Age, job satisfaction and diabetes were independent risk factors of hypertension [11]. Hypertension contributes to at least 45% of deaths due to heart diseases and 51% of deaths as a result of stroke [16]. In addition, it was found that there is an increase in physical inactivity, blood sugar level, alcohol use, and smoking among Nepalese residents in recent years [17]. A recent four-year analysis of the National Health Account showed that the highest healthcare spending was on NCDs at NPR 37.73 billion. Meanwhile, Out- of -Pocket (OOP) expenditure by disease and health conditions was highest for NCDs with 31% of OOP expenses [15]. A systematic review and meta-analysis conducted on prehypertension in Nepal revealed that prehypertension was significantly higher in male, middle aged adults and people from rural areas [18]. The discrepancy in the prevalence of prehypertension may have emerged due to diverse lifestyles, age difference, sex, educational level, job characteristics, urbanization, working conditions, cultures, and food choice habits.

In response to the prevention and control of NCDs in Nepal, the Package of Essential NCD Interventions (PEN) was adopted by the Ministry of Health and Population (MoHP) that aims to expand the coverage of essential services as well as essential medicines and diagnostics using the trained front line health workers [19]. Furthermore, the Government of Nepal adopted the Multisectoral Action Plan (2021–2025), National Health Policy 2019, and the target of SDGs (Sustainable Development Goals) 2030 to reduce premature death due to NCDs by one-third.

To the best of our knowledge, there are limited studies conducted on prehypertension and hypertension among government employees in Nepal. The relation between their mental workload and blood pressure is still under-explored. Government employees are a vital part of the nation’s workforce, primarily consisting of individuals in the working-age group. These employees have to face an increased workload, limited decision-making scope, lack of support from fellow workers, regular night shifts, limited physical mobility. All of these factors have a significantly influence prehypertension among employees [20]. Government agencies and stakeholders should closely consider and assess the health status of government employees.

This study on prehypertension and hypertension among government employees would assist policymakers in formulating effective strategies for screening, prevention, treatment, and control of hypertension and associated burden of disease. This study may provide additional evidence to identify high-risk groups and adopt relevant health promotion measures at the workplace aiming to control blood pressure and limit diseases caused by hypertension. Hence, this study aimed to describe the prevalence of prehypertension and its associated factors among government employees of Tilottama Municipality of Rupandehi district, Nepal.

## Materials and methodology

### Study design and setting

A cross-sectional study was conducted in Tilottama Municipality from 15 June 2023 to 10 November 2023. This municipality is located in the Rupandehi district of Lumbini Province, Nepal. It is one of the cities of Lumbini Province in Western Nepal. According to the 2021 Census, the total population of the city was 149,657, with a population density of 1185 km^2^ in 17 wards. The municipality has a literacy rate of 88.1% and there were nearly 36,000 households [21]. The prevalence of hypertension in Lumbini Province is 28.2% according to the STEPS survey, 2019.

### Study population

The study included government employees from 17 wards of Tilottama Municipality. It included the employees belonging to administrative and technical departments, health institutions and centers and school teachers working under the Government of Nepal either permanently or contract-based. Employees on leave, pregnant or ill at the time of study were excluded.

### Sample size and sampling technique

The sample size was calculated using the Cochrane single proportion formula n = Z^2^pq/d^2^ [22]. Assuming a 27% prevalence of prehypertension (p) from a similar study conducted by Agho et.al, 2018 in Nepal [4], 95% confidence interval (CI), 5% margin of error, and 10% non-response rate, the final sample size was determined as (n = 333) in this study.

A systematic random sampling technique was used to select participants for the study. A list of 900 government employees (N) was obtained from the administrative section *(Karmachari Prasasan)* of the municipal office. All participants were provided with a unique identity number. The first participant was chosen randomly using lottery method from the list of government employees. Then, participants were recruited at the regular interval of (N/n = 3^rd^). In the case of unavailability of any participant, the next corresponding participant was taken in the study. Out of 350 participants approached, responses were received from 333 participants, resulting in a non-response rate of 4.86%.

### Study tools

A study tool was obtained from the Nepal STEPs survey 2019 to assess prehypertension. A Nepali language tool was pretested to check the consistency and inter-reliability. The tool was pretested among 10% of the government employees of Sainamaina Municipality.

Section I: This section included questions regarding socioeconomic and demographic characteristics of the participants such as age (in completed years), sex, ethnicity, religion, education, type of employment and working department.

Section II: It consisted of questions related to behaviors and risk factors such as smoking, alcohol consumption, fruit and vegetable servings, and physical activities.

Section III: This section was related to occupational characteristics (duration of service, working hours, and stress level).

Part IV: This section consisted of questions regarding anthropometric measurements. It included measurement of participants’ height, weight, waist circumference, hip circumference, and blood pressure.

Participants’ height was measured with a portable standard stature tape and weight with a digital scale (SECA). Waist and hip circumference were measured using a non-stretchable tape. Participants’ blood pressure was measured using a digital automated blood pressure monitor (OMRON digital device) with a universal-size cuff.

### Data collection and techniques

A face-to-face interview was conducted using a structured questionnaire for data collection. Each interview took about 25–30 minutes including anthropometric and blood pressure measurements. Principal investigator and public health graduates were responsible for the data collection.

Blood pressure was measured using a digital automated blood pressure monitor (Omron Digital Device) equipped with a universal cuff size. To ensure accurate readings, participants rested for 15 minutes before blood pressure measurement using a digital blood pressure set. A total of 3 readings was taken, with a gap of 3 minutes in between each reading, and the mean of the 2nd and 3rd readings was taken for the analysis in this study.

Participants’ height was measured by using the following procedure; participants were asked to remove footwear and requested to stand on the flat surface with their feet together and knees straight. Weight was measured by using the following procedure; weighing machine was placed firmly on a flat surface, and then participants were asked to remove footwear with minimal possible clothes during measurement. Both height and weight were measured twice and the average values were computed in this study. Weight was measured using SECA scales (model SECA 874), and height was measured using the ShorrBoard® measuring board.

The waist circumference was taken at the midpoint between lower margin of last palpable rib and the top of iliac crest. Hip circumference was taken using non-stretchable tape, placing it at the widest part of the buttocks.

Screening of Diabetes Mellitus (DM) was conducted using capillary blood glucose (CBG) test and ACCU-CHECK strips which were analyzed by an ACCU-CHECK device standardized daily. The blood drops were collected on test strips and analyzed. Those participants who were found diabetic after random glucose tests were cross-verified through laboratory measurement.

Those individuals who were identified having hypertension and diabetic during the data collection were counseled and referred to Municipal Health Section of Tilottama Municipality.

### Data management and analysis

Collected data was systematically compiled, coded, edited and cross-checked in EpiData (version 3.1) before exporting to IBM SPSS (Statistical Package for Social Sciences) version 20.0 for analysis. The descriptive results were described in terms of number, percentages, mean and standard deviation. Bivariate analysis was performed using Chi-square tests. Multivariate logistic regression analysis was determined to assess the factors associated with prehypertension. A multicollinearity test was conducted among independent variables using variance inflation factor of cut-off-value < 5. Multiple regression models were developed using different variable inclusion criteria based on p-value thresholds (<0.1, < 0.2, and <0.3) and compared against a full model guided by theoretical relevance. The final multivariable logistic regression model included variables with p-values less than 0.2 to adjust for potential confounding factors. Model fit was evaluated using the Hosmer-Lemeshow goodness-of-fit test and the Nagelkerke R-squared statistic. Associations were considered statistically significant at a p-value of less than 0.05. Adjusted odds ratios (AORs) with 95% confidence intervals (CIs) were reported to reflect the strength and precision of the observed associations.

### Ethical considerations

The approval for the study was obtained from Institutional Review Committee (IRC) of B.P. Koirala Institute of Health Sciences (Reference number: 686/079/080-IRC). A letter of support was also obtained from the Tilottama Municipality Office to conduct the study. Both verbal and written informed consent were obtained from each participant. The interview was started after delineating study objectives. Confidentiality and privacy of participants was maintained and assured throughout the study.

### Operational definitions

#### Government employees.

Those employees who were selected through the Public Service Commission of Nepal or employees who have been working on a contract basis in the municipality. This study included employees from administrative and technical departments, health institutions and centers and school teachers.

#### Physical activity.

The metabolic equivalent of task (MET score) was calculated for every participant based on the information about the different activities and their duration. Based on the MET score, they were labelled as low-level (MET score<600), moderate-level (MET score between 600- < 3000) and high-level (MET score 3000 and above) [17].

#### Work-related stress.

The self-reported stress level of participants, categorized into three levels: “No stress”, “Some stress” and “High stress”.

#### Family history of hypertension or diabetes.

It was defined by the presence of hypertension or diabetes in parents, grandparents or siblings in their family of the participants.

#### Diabetes mellitus.

A random blood glucose level <140 mg/dl was considered normal, 140 mg/dL to <200 mg/dL was considered pre-diabetic, and ≥200 mg/dL was considered diabetic. Also, those individuals taking medication for diabetes as prescribed by health workers were considered diabetic [23].

#### Prehypertensive.

Based on the report of JNC 7 (Joint National Committee), an individual participant with systolic blood pressure 120–139 mm Hg and/or diastolic blood pressure 80–89 mm Hg is referred to as prehypertensive [3].

#### Hypertensive.

An individual having systolic BP (SBP) ≥140 mmHg or diastolic BP (DBP) ≥ 90 mmHg is referred to as hypertensive. Any individual on anti-hypertensive prescription medication at the time of the survey was also classified as hypertensive.

#### Normal blood pressure.

Individuals were classified as having a normal BP if SBP was < 120 mmHg and/or DBP < 80 mmHg.

#### Sufficient fruits and vegetables intake.

According to Food and Agriculture Organization (FAO) and WHO Expert consultation report, intake of at least 400 grams of fruits and vegetables in a day or at least 5 servings per day (80 gm/per serving) was regarded as sufficient.

#### Current smoker.

Current smoker was defined as those who reported smoking any tobacco product within the last 30 days.

#### Current alcohol drinker.

Those who consumed alcohol within the last 30 days was considered current alcohol drinkers.

#### Body Mass Index.

It was classified as underweight (<18.5 kg/m^2^), normal (18.5–24.99 kg/ m^2^), overweight (25–29.99 kg/m^2^) and obese (≥ 30 kg/m^2^) [24].

#### Waist-hip ratio.

The cut off point for normal value of waist-hip ratio for females and males was taken as <0.8 cm and <0.9 cm respectively [17].

## Results

### Individual characteristics of the participants

A total of 333 employees participated in this study. The mean (± SD) age of the participants was 36.9 (± 8.8) years. About (54.1%) of participants were females and majority (85.3%) of participants were married. The majority of them were Hindu in religion (96.7%) and Brahmin/Chhetri in ethnicity (85.3%). About 39% of the participants had completed at least graduate-level education. Around half of them (52.9%) were permanent government employees, while, 50.5% and 24% belonged to school teachers and administrative departments respectively (Table 1).

**Table 1:** Individual characteristics of participants.

Characteristics	Number (n)	Percentage (%)
**Age (in years) Mean ± SD**	**36.9 ± 8.8**	
<30	70	21.0
30-39	150	45.0
40-49	74	22.3
50-59	39	11.7
**Sex**		
Male	153	45.9
Female	180	54.1
**Marital status**		
Married	284	85.3
Unmarried	43	12.9
Widowed	6	1.8
**Religion**		
Hindu	322	96.7
Buddhist	11	3.3
**Ethnicity**		
Brahmin/Chettri	284	85.3
Janajati	43	12.9
Others (Madhesi, Dalit)	6	1.8
**Education level completed**		
Primary level (class 1–8)	10	3.0
Secondary level (class 9–12)	80	24.0
Graduate level	129	38.8
Masters level and above	114	34.2
**Type of family**		
Joint/extended	177	53.2
Nuclear	156	46.8
**Type of employment**		
Permanent	176	52.9
Contract based	157	47.1
**Working department**		
Teacher	168	50.5
Administrative	80	24.0
Health	61	18.3
Technical (other than health)	24	7.2

### Anthropometric, blood pressure and blood glucose level measurements of participants

This study found that nearly one-third (29.4%) of the participants were prehypertensive and a considerable portion (14.1%) were hypertensive. Among hypertensive participants, 6.4% were pre-diabetic and 10.6% were diabetic when random blood glucose was measured. This study found that 46.8% and 13.6% of the participants were overweight and obese respectively. Two-thirds of the males (65.4%) had a waist-hip ratio equal or more than 0.9 meanwhile 60.6% female had a waist-hip ratio equal to or more than 0.85 (Table 2).

**Table 2:** Anthropometric, blood pressure and blood glucose level measurements of the study participants.

Characteristics	Number(n)	Percentage (%)
**Ever had BP measured by health workers**		
Yes	302	90.7
No	31	9.3
**Blood pressure status**		
Normal	188	56.5
Prehypertension	98	29.4
Hypertension	47	14.1
**Status of the level of blood glucose among hypertensive (n = 47)**		
Normal	39	83.0
Pre-diabetic	3	6.4
Diabetic	5	10.6
**BMI (kg/m** ^**2)**^		
**Mean ± SD**	25.9 ± 3.7	
Underweight	8	2.4
Normal	124	37.2
Overweight	156	46.8
Obese	45	13.6
**Waist-hip ratio of male (n = 153)**		
≥ 0.9	100	65.4
<0.9	53	34.6
**Waist -hip ratio of female (n = 180)**		
≥ 0.85	109	60.6
<0.85	71	39.4
**Waist hip ratio**		
Obese	209	62.8
Normal	124	37.2

### Behavioral and risk factors of prehypertension

Table 3 outlines the risk factors associated with prehypertension among the study participants. About 7.5% of participants were ever smokers and the initiation age of smoking was 22 years. About (14.7%) were current alcohol users and 3.3% were current smokers. The intake of fruits and vegetables according to the recommended servings of WHO was very low (1.2%).

**Table 3:** Behavioural and risk factors of prehypertension among the study participants.

Characteristics	Number (n)	Percentage (%)
**Ever smoker**		
Yes	25	7.5
No	308	92.5
**Initiation age of smoking (in years)**		
**Mean± SD**	22.24 ± 5.72	
**Current smoker**		
Yes	11	3.3
No	322	96.7
**Ever consumed alcohol**		
Yes	97	29.1
No	236	70.9
**Current alcohol user**		
Yes	49	14.7
No	284	85.3
**Consumption of fruits and vegetable (serving/day)**		
≥5 servings	4	1.2
<5 servings	329	98.8
**Type of oil used in cooking**		
Mustard oil	238	71.5
Refined vegetable oil	93	27.9
Butter /ghee	2	0.6
**Salt adding habit**		
Always	5	1.5
Often	15	4.5
Sometimes	91	27.3
Rarely	80	24.0
Never	142	42.6
**Consumption of processed food containing high salt**		
Always		
Often	20	6.0
Sometimes	173	52.0
Rarely	110	33.0
Never	30	9.0
**Perceived importance of lowering salt intake**		
Very important	184	55.3
Somewhat important	144	43.2
Not at all	5	1.5
**Vigorous intensity activities**		
Yes	96	28.8
No	237	71.2
**Moderate intensity activities**		
Yes	194	58.3
No	139	41.7
**Yoga/Meditation**		
Yes	96	28.8
No	237	71.2
**Level of physical activity (MET- minute per week)**		
<600	150	45.0
≥ 600	183	55.0
**Family history of hypertension**		
Yes	162	48.6
No	171	51.4
**Family history of diabetes**		
Yes	92	27.6
No	241	72.4

The participants involving in vigorously and moderately intense activities were 28.8% and 58.8% respectively. Among those participants who were engaged in physical activity, 55% had 600 or more MET-minutes per week indicating adequate physical activity. About 48.6% of participants had a family history of hypertension, and 27.6% had family history of diabetes.

### Occupational characteristics and stress level among participants

This study shows that more than 70% of the participants had been involved in government service for at least 5 years. Meanwhile, 18.3% and 22.2% of the participants worked more than 48 hours weekly and had their sleep duration of less than 7 hours per day respectively. More than half (53.5%) expressed that they had some level of work-related stress. Nearly one in three participants had stressful life events in the past. (Table 4)

**Table 4:** Occupational characteristics and stress level among participants.

Characteristics	Number (n)	Percentage (%)
**Duration of service (in years)**		
<5	100	30.0
5-9	98	29.5
10-19	84	25.2
≥20	51	15.3
**Working hours (hours in a week)**		
≤48 hours	272	81.7
> 48 hours	61	18.3
**Sleep duration (hours per day)**		
<7 hours	74	22.2
≥ 7 hours	259	77.8
**Work-related stress**		
No	139	41.7
Some	178	53.5
High	16	4.8
**Stress at home**		
No	137	41.1
Some	190	57.1
High	6	1.8
**Stressful life events in past years**		
Yes	98	29.4
No	235	70.6

### Morbid conditions among participants (self-reported)

This study showed that some participants suffered from morbid conditions such as diabetes (6.0%), thyroid disorder (5.7%), dyslipidaemia (4.8%), and COPD (2.1%) (Table 5).

**Table 5:** Prevalence of morbid conditions (self-reported) among participants.

Characteristics	Number (n)	Percentage (%)
**Diabetes mellitus**		
Yes	20	6.0
No	313	94.0
**COPD**		
Yes	7	2.1
No	326	97.9
**Dyslipidemia**		
Yes	16	4.8
No	317	95.2
**Thyroid disorder**		
Yes	19	5.7
No	314	94.3
**Others (osteoarthritis, anemia, back pain)**		
Yes	6	1.8
No	327	98.2

### Factors associated with the prevalence of prehypertension

In bivariate analysis, the association of prehypertension with various predictors was assessed excluding the 47 hypertensive participants, resulting in a total of 286 total observations. The factors associated with prehypertension in the bivariate logistics regression analysis were age, sex, working department, type of family, family history of hypertension, BMI, waist-hip ratio, ever alcohol use, and duration of service.

Multivariate analysis was used to determine the factors associated with the prevalence of prehypertension. This study showed that participants working in non-health departments had 3.2 times higher odds of prehypertension compared to those in health departments (AOR = 3.2, 95% CI: 1.4–7.0, p = 0.003), those with a family history of hypertension had 1.9 times higher odds compared to those without such a history (AOR = 1.9, 95% CI: 1.1–3.4, p = 0.019), and individuals with BMI ≥ 25 had 1.7 times higher odds compared to those with BMI < 25 (AOR = 1.7, 95% CI: 1.0–3.1, p = 0.049) (Table 6).

**Table 6:** Binary logistic regression to determine the predictors of prehypertension (n = 286).

Characteristics	Prehypertension		COR, 95% CI	p-value	AOR, 95% CI	p-value
	Yes n (%)	No n (%)				
	98(34.3)	188 (65.7)				
**Age**						
<40	59(29.1)	144(70.9)	Ref	**0.004**	Ref	0.427
≥40	39(47.0)	44(53.0)	2.1(1.2-3.6)		1.3(0.6-2.6)	
**Sex**						
Female	45(28.3)	114(71.7)	Ref	**0.018**	Ref	0.172
Male	53(41.7)	74(58.3)	1.8(1.1-2.9)		1.5(0.8-3.0)	
**Type of family**						
Nuclear	50(38.5)	80(61.5)	Ref	0.173	Ref	0.233
Joint	48(30.8)	108(69.2)	1.4(0.8-2.2)		1.3(0.8-2.4)	
**Working department**						
Health	11(20.0)	44(80.0)	Ref	**0.015**	Ref	0.003*
Non-health	87(37.7)	144(62.3)	2.4(1.1-4.9)		3.2(1.4-7.0)	
**Family history of hypertension**						
No	44(28.0)	113(72.0)	Ref	**0.015**	Ref	0.019*
Yes	54(41.9)	75(58.1)	1.8(1.1-3.0)		1.9(1.1-3.4)	
**Family history of diabetes**						
No	68(32.1)	144(67.9)	Ref	0.188	Ref	0.249
Yes	30(40.5)	44(59.5)	1.4(0.8-2.4)		1.4(0.7-2.6)	
**BMI**						
<25	31(25.4)	91(74.6)	Ref	0.008	Ref	0.049*
≥ 25	67(40.9)	97(59.1)	2.0(1.2-3.3)		1.7(1.0-3.1)	
**Waist hip ratio**						
Normal	29(25.0)	87(75.0)	Ref	0.007	Ref	0.073
Obese	69(40.6)	101(59.4)	2.0(1.2-3.4)		1.7(0.9-3.0)	
**Ever alcohol user**						
Yes	34(43.6)	44(56.4)	Ref	0.043	Ref	0.487
No	64(30.8)	144(69.2)	1.7(1.0-2.9)		1.2(0.6-2.5)	
**Duration of service**						
<10 years	51(28.0)	131(72.0)	Ref	**0.004**	Ref	0.236
10 years and above	47(45.2)	57(54.8)	2.1(1.2-3.5)		1.5(0.7-2.9)	

* Statistically significant at p-value<0.05,

*Ref = Reference.*

## Discussion

This study assessed the prevalence of prehypertension and its associated factors among government employees and found that the prevalence of prehypertension was 29.4% among government employees. This finding is similar to the national survey of Nepal (29.4%), and a study conducted by Agho et.al, 2018(26.9%) [4,17]. In contrast, a study carried out in North East India showed a higher prevalence of prehypertension (40.8%) which is higher than our study [25]. The low prevalence in our study, compared to others, might be because we could not cover participants aged 60 and above due to the Nepal Government’s retirement protocol. This age group is considered to be at higher risk. However, one of the studies conducted at Tertiary Hospital in Kathmandu showed a higher prevalence of prehypertension (67.4%) among health workers [20]. This high prevalence may be due to dual responsibility towards work and family, night shifts among health workers which force them to practice unhealthy behaviours, and a sedentary lifestyle which ultimately leads to high blood pressure.

This study demonstrated that age was not associated with the prevalence of prehypertension. However, studies done in Bangladesh [26], India [27], China [28] and Nepal [4] showed contrary results to this study. It might be because most of our participants were around 40 years old because of the policy of Government of Nepal in which government employees retire before the age of 60. Regarding sex, in our study the prevalence of prehypertension was higher (41.7%) among male compared to female which is similar to another study conducted in different parts of Nepal by Agho et. al. [4], Ghimire et. al. [20], Tamrakar et. al [29]. These findings are also consistent with nationwide STEPS Survey 2019 and Nepal Demographic Health Survey 2022 [17,30]. This is due to biological differences such as sex hormones, chromosomal differences, and other biological sex differences that are protective against high blood pressure in women [31,32].

The odds of prehypertension was about 1.9 times among employees who had a family history of hypertension. This finding is in line with the studies conducted in China [28], India [27] and Malaysia [33]. Individuals with a FH of hypertension form an easily identifiable group who may benefit from targeted interventions [34]. Thus, early identification of prehypertension among employees with a family history of hypertension will be useful to help advise “risky individuals” for more frequent monitoring of BP as an early intervention.

This study demonstrated that employees who belonged to non-health departments had higher odds of prehypertension (AOR = 3.2) than their counterparts. It may be because government health workers are more conscious of their health and apply preventive measures compared to other employees. Another reason could be due to the higher health literacy among health workers than non-health workers.

Overweight and obesity are significant determinants of hypertension. This study found that abnormal BMI was associated (AOR = 1.7) with prehypertension and is coherent with the studies conducted in Nepal [35,36]. It is supported by a study conducted in Asia which reported that overweight increases the risk of hypertension by 94% [37]. The higher body mass index increases the risk of elevated blood pressure include increased resistance to insulin and retention of salt [38] and decreased physical activity [39].

Waist-to-hip ratio is considered as the better predictor of abdominal obesity [40]. This study reported that more than half of the employees had abdominal obesity. However, this result is contrary to the studies conducted among bankers in Bangladesh and police personnel in India [35,36].

This study found no association between duration of service and prehypertension. However, the study in West Bengal among police personnel revealed a significant association between duration of service with the prehypertension [35].

It was found that stress causes hypertension through repeated blood pressure elevation as well as by stimulation of the nervous system producing large amounts of vasoconstricting hormone that increase blood pressure [41]. Contrary to this study, our study reflected that stress was not associated with prehypertension.

Our study showed a very low fruit and vegetable consumption among the government employees**.** A similar pattern was found in the STEPS survey of Nepal which showed that 96.7% of adults had less than five servings of fruits and vegetables per day. The fluctuating pattern of fruit and vegetables consumption in Nepal due to the seasonal availability of these foods [42].

Alcohol drinking is considered as a risk factor for prehypertension. Various evidences suggest that there was a direct association between higher alcohol consumption and prehypertension [43]. However, this finding demonstrated no association between alcohol drinking and prehypertension. It is probably due to a smaller number of alcohol users that could influence the results in this study.

Despite having good educational background, many of the government employees did not practice any physical activity. Very less proportion of employees reported being involved in physical activity in this study. It might be due to busy schedules, working environment and poor work-life balance of employees. Therefore, public health awareness programs should be expanded targeting both private and government employees to improve physical activity, working environment and healthy behaviour to minimize the risk of hypertension [44].

### Implications to practice

Local governments should collaborate with private organizations and development partners to establish comprehensive hypertension prevention and control programs. This should include regular blood pressure screenings, particularly targeting government employees, as a proactive measure for the early detection of prehypertension. Additionally, institutions should integrate workplace wellness initiatives, such as stress management workshops, physical activity programs, and the provision of healthier food options in cafeterias.

### Limitations

Although this study provides valuable insights into prehypertension prevalence among urban government employees, several limitations must be acknowledged. This study is a cross-sectional study so it cannot show causality. There might have been some bias in self-reported behaviors such as tobacco use, alcohol use, physical activity and perceived stress levels including recall bias regarding long-term habits. The study was conducted among urban government employees due to which the result may not be generalized to all government employees/municipalities of Nepal. Also, the lack of interaction analysis between occupational stress and other risk factors was also not explored. A deeper understanding of the specific tasks, workload, and physical demands within each department would have provided more nuanced data and enhanced our ability to identify departmental-specific risk factors.

## Conclusion

This study concluded that a significant proportion of government employees had prehypertension and hypertension. Working department, family history of hypertension, and BMI were found to be statistically significant with prehypertension.

This study findings suggest the imperative for targeted interventions tailored to mitigate specific risks associated with varying occupational contexts, family history, and BMI. Implementation of workplace health programs, provision of physical activity, healthy food choices and dietary practices should be promoted targeting all employees. Regular hypertension screening program and public health interventions should be promoted among targeted population to prevent and control hypertension. Future research could benefit from longitudinal studies or randomized controlled trials to assess causality and provide deeper insights into the relationships observed.

## Supporting information

S1 File Study data file. (CSV)

S1 File Study Questionnaire. (DOCX)
