# Supplementary material for: Prehypertension and its associated factors among government employees in Tilottama Municipality of Rupandehi District, Nepal
Source: PLoS One. 2025 Dec 11;20(12):e0338625. doi: 10.1371/journal.pone.0338625 (PMC12697992; doi:10.1371/journal.pone.0338625)
Supplement: S1 File — (DOCX) [file pone.0338625.s002.docx]

**Participant Information Sheet**

**Namaste!**

I am Sheetal Bhandari, a student of School of Public Health and Community Medicine at B.P. Koirala Institute of Health Sciences, Dharan, conducting research under the supervision of my guide, Prof. Dr. Paras Kumar Pokharel. The title of my research is “Prehypertension and its predictors among government employees in Tilottama Municipality, Rupandehi”.

I am going to give you information and invite you to be a part of this research. You do not have to decide today whether or not you will participate in the research. Before you decide, you can talk to anyone you feel comfortable about the research.

There may be some words that you do not understand. Please ask me to stop as we go through the information and I will take time to explain. If you have questions later, you can ask them to me at any time.

**Purpose of the research**

To estimate the prevalence of prehypertension and associated factors among government employees of Tilottama Municipality, Rupandehi

**Participant selection**

Government employees working in Tilottama Municipality

**Voluntary participation**

Your participation in this research is entirely voluntary. It is your choice whether to participate or not.

**Procedures and protocol**

A direct interview will be conducted through a semi-structured questionnaire. During data collection, weight and height will be taken using different instruments. Along with it, blood pressure measurement of participants will also be taken to determine prehypertension. The interview will last approximately 25 to 30 minutes.

**Confidentiality**

Any personal information that we collect from this research project will be kept confidential. There will be freedom of individual to participate and to withdraw from research at any time without penalty or loss of benefits to which the subject would otherwise be entitled.

**Sharing the results**

The knowledge that we get from this research will be published in a thesis paper and later may be published in a scientific research journals.

For further information, please contact

Sheetal Bhandari
Mobile no: +977 9867710846

Email id: [sheetal.bhandari07@gmail.com](mailto:sheetal.bhandari07@gmail.com)

This proposal has been reviewed and approved by the Institutional Review Committee of School of Public Health and Community Medicine at B.P. Koirala Institute of Health Sciences, Dharan, which is a committee whose one of the major tasks is to make sure that research participants are protected from harm.

**Participant Consent Form**

I am being invited to participate in a research titled “**Prehypertension and its predictors among government employees in Tilottama Municipality, Rupandehi”**. The content of the information sheet provided has been read carefully by me /has been explained to me in detail, in a language that I comprehend, and have fully understood the contents. The nature and purpose of the study and its potential risks/ benefit and expected duration of the study, and other relevant details of the study have been explained to me in detail. I understand that my participation is voluntary and that I am free to withdraw at any time, without giving any reason. I hereby provide my consent to take part in the above study. I understand that information obtained from me, might be used for the purpose of publication in textbooks or medical journals and dissertation purposes, and/or for medical education. The consent form has been signed by me in full consciousness when I was not under the influence of any drugs or pressure of any kind.

Respondent’s signature _____________

Researcher’s signature ______________

**Questionnaire**

|  | | | |
| --- | --- | --- | --- |
| **S.N.** | **Question** | **Response** | **Code** |
| 1. | Age | In Completed years…… | C1 |
| 2. | Sex | Male 1  Female 2 | C2 |
| 3. | Marital Status | Never Married 1  Married 2  Divorced 3  Widowed 4 | C3 |
| 4. | Caste/Ethnicity | Brahmin/ Chhetri 1  Janajati 2  Dalit 3  Madhesi 4  Muslim 5  Other (Specify) 6 | C4 |
| 5. | Religion | Hindu 1  Buddhist 2  Muslim 3  Kirat 4  Christian | C5 |
| 6. | Highest level of education completed | Informal Education 1  Basic level (class 1-8) 2  Secondary level (class 9-12) 3  Graduate 4  Masters or above 5 | C6 |
| 7. | Type of Family | Nuclear 1  Joint 2  Extended 3 | C7 |
| 8. | Annual Family Income | NRs….. | C8 |
| 9. | Years of experience | No. of years | C9 |
| 10. | Type of government employee | Permanent 1  Contract based 2 | C10 |
| 11. | Level | Non-gazette employee 1  4^th^ level 2  5^th^ level 3  6^th^ level 4  7^th^ level 5  8^th^ level 6  9^th^ level 7  10^th^ level 8 | C11 |
| 12. | Department | Administrative 1  Teacher……………subject….. 2  Health 3  Technical (other than health ) 4 | C12 |
| 13. | Working Hours | ≤ 48 hours 1  >48 hours 2 | C13 |

| **14.Blood Pressure Measurement** | | | |
| --- | --- | --- | --- |
| a. | Reading 1 | Systolic BP (mm Hg) |  |
|  |  | Diastolic BP (mm Hg) |  |
| b. | Reading 2 | Systolic BP (mm Hg) |  |
|  |  | Diastolic BP (mm Hg) |  |
| c. | Reading 3 | Systolic BP (mm Hg) |  |
|  |  | Diastolic BP (mm Hg) |  |
| d. | Mean of Readings |  |  |
| e. | Random Blood Glucose (mg/dl) |  |  |
| **Physical Measurement** | | | |
| f. | Height (cm) |  |  |
| g. | Weight(kg) |  |  |
| h. | Hip circumference (cm) |  |  |
| i | Waist circumference (cm) |  |  |

| **Behavioral Characteristics( Tobacco use, Alcohol consumption , Diet)** | | | |
| --- | --- | --- | --- |
| **S.N.** | **Question** | **Response** | **Code** |
| 15. | Do you smoke any tobacco products?(e.g. cigratees, bidi, hukah etc)  (USE SHOWCARDS) | Yes 1  No 2 (if no skip to A1)  In past 3 | T1 |
| 16 | Do you currently smoke tobacco products daily? | Yes 1  No 2 | T2 |
| 17 | How old were you when you first started smoking? | Age (in years) | T3 |
| 18 | Do you remember how long it was? | In years…  In months…..  In weeks….. | T4 |
| 19 | Do you currently use any smokeless tobacco products such as snuff, chewing tobacco, Khaini ,gutka etc?(USE SHOWCARDS) | Yes 1  No 2  In past 3 | T5 |
| 20 | During the past 12 months, have you tried to **stop smoking?** | Yes 1  No 2 | T6 |
| 21 | During any visit to a doctor or other health worker in the past 12 months, were you advised to quit smoking tobacco? | Yes 1  No 2  No visit during the past 12 months 3 | T7 |
| 22 | Do you currently use smokeless tobacco products daily? | Yes 1  No 2 | T8 |
| 23 | On average, how many times a day/week do you use smoke tobacco products | No of times…….daily  No of times ……weekly | T9 |
| 24 | How many times a day/week do you use smokeless tobacco products? | No of times…….daily  No of times ……weekly | T10 |

| **Alcohol Consumption** | | | |
| --- | --- | --- | --- |
| 25 | Have you ever consumed any alcohol such as beer, wine, spirits (add other local examples)?  (USE SHOWCARDS) | Yes 1  No 2 (if no skip to D1)  In past 3 | A1 |
| 26 | Have you consumed any alcohol within past 12 months? | Yes 1  No 2 (if no go to D1) | A2 |
| 27 | During the past 12 months, how frequently have you had at least one standard alcoholic drink?(READ RESPONSES ,USE SHOWCARDS) | Daily 1  5-6 days per week 2  3-4 days per week 3  1-2 days per week 4  1-3 days per month 5  Less than once a month 6  Never 7 | A3 |
| 28 | Have you consumed any alcohol within past 30 days? | Yes 1  No 2 (If no go to F1) | A4 |
| 29. | During the past 30 days, on how many occasions did you have at least one standard alcoholic drink? | Number…… | A5 |
| 30. | During the past 30 days, what was the **largest number of standard drinks** you had on a single occasion, counting all types of alcoholic drinks together? | Largest number…… | A6 |
| 31. | During each of the past 7 days, how many standard drinks did you have each days?  (USE SHOWCARDS) | Sunday  Monday  Tuesday  Wednesday  Thursday  Friday  Saturday | A7 |

| **Diet** | | | |
| --- | --- | --- | --- |
| 32. | In a typical week, on how many days do you eat fruit?  (USE SHOWCARDS) | No of days.. | D1 |
| 33. | How many servings of fruit do you eat on one of those days?  (USE SHOWCARDS) | No of servings | D2 |
| 34. | In a typical week, on how many days do you eat vegetables?  (USE SHOWCARDS) | No of days | D3 |
| 35 | How many servings of vegetables do you eat on one of those days?  (USE SHOWCARDS) | No of servings…. | D4 |
| 36 | What type of oil or fat is most often used for meals preparation in your household? | Mustard oil 1  Refined vegetable oil 2  Vansapati ghee 3  Butter ghee 4  Lard or suet 5  Other (specify) 6 | D5 |
| **Dietary salt** | | | |
| 37 | How often do you add salt or salty sauce to your food right before you eat it or as you are eating it?  (select only one)  (USE SHOWCARDS) | Always 1  Often 2  Sometimes 3  Rarely 4  Never 5  Don’t Know 6 | D6 |
| 38 | How often is salt, salty seasoning or a salty sauce added in cooking or preparing foods in your households? | Always 1  Often 2  Sometimes 3  Rarely 4  Never 5  Don’t Know 6 | D7 |
| 39 | How often do you eat processed food high in salt? By processed food high in salt means food that have been altered their natural state, such as packaged, salty snacks, canned salty food, papad, tilauri, salty food prepared at a fast food restaurants, chauchau ,lays etc.?  (USE SHOWCARDS) | Always 1  Often 2  Sometimes 3  Rarely 4  Never 5  Don’t Know 6 | D8 |
| 40 | How important to you is lowering salt intake in your diet? | Very important 1  Somewhat important 2  Not at all 3  Don’t know 4 | D9 |
| 41 | Do you think that too much salt or salty sauce in your diet would cause a health problem? | Yes 1  No 2 | D10 |
| 42 | Do you do any of the following on a regular basis to control your salt intake?  (RECORD FOR EACH) |  | D11 |
| a | Limit consumption of processed foods | Yes 1  No 2 |  |
| b | Look at the salt or sodium content on food labels | Yes 1  No 2 |  |
| c | Buy low salt/sodium alternatives | Yes 1  No 2 |  |
| d | Use spices other than salt when cooking | Yes 1  No 2 |  |
| e | Avoid eating foods prepared outside of a home | Yes 1  No 2 |  |
| f | Do other things specifically to control your salt intake | Yes 1  No 2 |  |
| g | Other (Please specify) |  |  |

| **Dietary Diversity Questionnaire** | | | |
| --- | --- | --- | --- |
| S.N. | Food categories | Haven you eaten these following foods in the past 24 hours? | Code |
| 43. | Cereals(Rice, Millet, Dhido, Roti), Wheat/ Buckwheat, Corn Dhido/ Roti/ Bhat  Prridge/oat bran/oat meal | Yes 1  No 2  Don’t know 3 | E1 |
| 44. | White roots and tubers  Potato ,yam, turnip | Yes 1  No 2  Don’t know 3 | E2 |
| 45. | Pulses (beans, peas and lentils)  (Soyabean, chickenpea, black gram, green gram, French bean, peas (dry), lentil (dal, pulses) | Yes 1  No 2  Don’t know 3 | E3 |
| 46. | Nuts and Seeds  Almond, Peanut,walnut,cashew,pumpkin seed, sunflower seed,sunflower seed | Yes 1  No 2  Don’t know 3 | E4 |
| 47. | Milk and milk products |  | E5 |
| a. | Milk as a  drink | Yes 1  No 2  Don’t know 3 |  |
| b. | Yoghurt or other milk products (cheese,panner) | Yes 1  No 2  Don’t know 3 |  |
| 48. | Meat poultry and fish |  | E6 |
| a | Any meat such as goat,lamb, chicken, pork,buff, ducks | Yes 1  No 2  Don’t know 3 |  |
| b | Any organ meat such as liver, kidney | Yes 1  No 2  Don’t know 3 |  |
| c | Fresh or dried fish | Yes 1  No 2  Don’t know 3 |  |
| 49. | Eggs (Chicken eggs, duck eggs) | Yes 1  No 2  Don’t know 3 | E7 |
| 50. | Dark green leafy vegetables (Amaranth leaves, Mustard leaves, Spinach, Broccoli, Pumpkin greens, Coriander leaves) | Yes 1  No 2  Don’t know 3 | E8 |
| 51. | Vitamin A rich vegetables and fruits (carrots, pumpkin, squash (orange or drank yellow fleshed only),sweet potato ,mango , papaya | Yes 1  No 2  Don’t know 3 | E9 |
| 52. | Other vegetables(brinjal, cabbage, cauliflower, cucumber, Mushroom, Onion, Radish, Tomato, Parwal, Bitter gourd) | Yes 1  No 2  Don’t know 3 | E10 |
| 53. | Other fruits (Apple, banana ,Guava, pear, pomegranate, pineapple) | Yes 1  No 2  Don’t know 3 | E11 |
| 54. | Other processed food |  | E12 |
| a. | Biscuits, plain sweets, chocolate, noodles, pasta, cake, cold drinks, momo /chowein, ice-cream | Yes 1  No 2  Don’t know 3 |  |
| b. | Others (please specify foods which are not in list ,but consumed in last 24 hour) |  |  |
| c. | Was last day a special day, like a celebration birthday, feast day, fasting, sickness etc. in which you ate special food or more or less than usual or did not eat because of fasting? | How many times have you eaten ………? In the past day |  |

| **Physical Activities** | | | |
| --- | --- | --- | --- |
| **S.N.** | **Question** | **Response** | **Code** |
| 55. | Does your work involve Vigorous intensity activity? (Causes large increase in breathing/heart rate eg: carrying or lifting heavy loads, digging, ploughing, cycling rikshaw, construction work for at least 10 minutes continuously)  (USE SHOWCARD) | Yes 1  No 2 | P1 |
| 56. | In a typical week, on how many days do you do vigorous intensity activities as part of your work? | No of days…… | P2 |
| 57. | How much time do you spend doing vigorous intensity activities on a typical day? | Hours: Min.... | P3 |
| 58. | Does your work involve Moderate intensity activity that causes small increase in breathing/heart rate eg: brisk walking, carrying light loads, manual washing clothes, mopping of floor, gardening at home for at least 10 minutes continuously?  (USE SHOWCARD) | Yes 1  No 2 | P4 |
| 59. | In a typical week, on how many days do you do moderate intensity activities as part of your work? | No of days…… | P5 |
| 60. | How much time do you spend doing moderate intensity activities at work on a typical day? | Hours: Min ... | P6 |
| 61. | Sedentary behavior (how much time do you usually spend sitting or reclining on a typical day? | Hours: Min ... | P7 |
| 62. | Do you practice yoga, pranayama or meditation? | Yes 1  No 2 | P8 |

| **Mental health (Subjective report)** | | | |
| --- | --- | --- | --- |
| **S.N** | **Question** | **Response** | **Code** |
| Do you have any of the following stress? | | | |
| 63. | Work /business stress | No 1  Some 2  High 3 | M1 |
| 64. | General stress at home | No 1  Some 2  High 3 | M2 |
| 65. | Severe financial stress/due to unemployment | Yes 1  No 2 | M3 |
| 66. | Stressful life events in past years which disturbed you a lot | Yes 1  No 2 | M4 |
| 67. | Sleep duration |  |  |

| **History of Raised Blood Pressure** | | | |
| --- | --- | --- | --- |
| 68. | Do you have family history of hypertension? | Yes 1  No 2 |  |
| 69. | Have you ever had your blood pressure measured by a doctor or other health worker? | Yes 1  No 2(if no skip to next part) |  |
| 70 | Have you ever been told by a doctor or other health worker that you have a raised BP or hypertension? | Yes 1  No 2 |  |
| 71. | Were you first told in the past 12 months? | Yes 1  No 2 |  |
| 72. | In past two weeks, have you taken any drugs (medication) for raised blood pressure prescribed by a doctor or other health worker? | Yes 1  No 2 |  |
| 73. | Where do you usually go when you have health problem? | Govt Hospital 1  Govt PHC/HP 2  Community Hospital/NGO run 3  Private Hospital 4  Private Clinic 5  Pharmacy 6  Ayurvedic, Homeopathy /Naturopathy 7 |  |
| 74. | Have you ever seen a traditional healer like Dhani/Jhakri/Purohit/Lama/ Gubaju/ Matas for raised blood pressure or hypertension? | Yes 1  No 2 |  |
| 75. | Are you currently taking any herbal or traditional remedy for your raised blood pressure? | Yes 1  No 2 |  |

| **History of Diabetes** | | | |
| --- | --- | --- | --- |
| S.N. | Question | Response | Code |
| 76. | Do you have family history of diabetes? | Yes 1  No 2 | D1 |
| 77. | Have you ever had your blood sugar checked by a doctor or other healthcare professional? | Yes 1  No 2  (if no skip to que no I1) |  |
| 78. | Have you ever been told by a doctor or other health worker that you have a raised blood sugar or diabetes? | Yes 1  No 2  (if no skip to que no I1) | D2 |
| 79. | Were you first told in the past 12 months? | Yes 1  No 2 | D3 |
| 80. | Are you currently taking insulin for diabetes prescribed by a doctor or other health worker? | Yes 1  No 2 | D4 |
| 81. | In past two weeks, have you taken any drugs (medication) for diabetes prescribed by a doctor or other health worker? | Yes 1  No 2 | D5 |
| 82. | Have you ever seen a traditional healer for diabetes or raised blood sugar? | Yes 1  No 2 | D6 |
| 83. | Are you currently taking any herbal or traditional remedy for your diabetes? | Yes 1  No 2 | D7 |

| **Other illness** | | | | | |
| --- | --- | --- | --- | --- | --- |
| S.N | Disease condition | Response | Duration | Medication | Code |
| 84. | Diabetes mellitus | Yes 1  No 2 |  | Taking  Not taking | I1 |
| 85. | COPD | Yes 1  No 2 |  | Taking  Not taking | I2 |
| 86. | Dyslipidemia | Yes 1  No 2 |  | Taking  Not taking | I3 |
| 87. | Thyroid disorders | Yes 1  No 2 |  | Taking  Not taking | I4 |
| 88. | Other | Yes 1  No 2 |  | Taking  Not taking | I5 |

| **Lifestyle Advice** | | | |
| --- | --- | --- | --- |
| S.N. | Question | Response | Code |
| 89. | During the past 12 months, have you visited a doctor or other health worker? | Yes 1  No 2 | L1 |
| 90. | During any of your visits to a doctor or other health worker in the past 12 months, were you advised to do any of the following?  (RECORD FOR EACH) | | |
| a | Reduce salt in your diet | Yes 1  No 2 | L2 |
| B | Quit using tobacco or don’t start | Yes 1  No 2 |  |
| C | Eat at least five servings of fruit and /or vegetables each day | Yes 1  No 2 |  |
| d | Reduce fat in your diet | Yes 1  No 2 |  |
| e | Start or do more physical activity | Yes 1  No 2 |  |
| f | Maintain a healthy body weight or lose weight | Yes 1  No 2 |  |
| g | Reduce sugary beverages in your diet | Yes 1  No 2 |  |

Thank you for your participation!
